# Oxidative and glycolytic skeletal muscles show marked differences in gene expression profile in Chinese Qingyuan partridge chickens

**DOI:** 10.1371/journal.pone.0183118

**Published:** 2017-08-16

**Authors:** Shu Jingting, Xiao Qin, Shan Yanju, Zhang Ming, Tu Yunjie, Ji Gaige, Sheng Zhongwei, Zou Jianmin

**Affiliations:** Key laboratory for poultry genetics and breeding of Jiangsu province, Institute of Poultry Science, Chinese Academy of Agricultural Science, Yangzhou, Jiangsu, China; University of Maryland Center for Environmental Science, UNITED STATES

## Abstract

Oxidative and glycolytic myofibers have different structures and metabolic characteristics and their ratios are important in determining poultry meat quality. However, the molecular mechanisms underlying their differences are unclear. In this study, global gene expression profiling was conducted in oxidative skeletal muscle (obtained from the *soleus*, or SOL) and glycolytic skeletal muscle (obtained from the *extensor digitorum longus*, or EDL) of Chinese Qingyuan partridge chickens, using the Agilent Chicken Gene Expression Chip. A total of 1224 genes with at least 2-fold differences were identified (*P* < 0.05), of which 654 were upregulated and 570 were downregulated in SOL. GO, KEGG pathway, and co-expressed gene network analyses suggested that *PRKAG3*, *ATP2A2*, and *PPARGC1A* might play important roles in myofiber composition. The function of *PPARGC1A* gene was further validated. *PPARGC1A* mRNA expression levels were higher in SOL than in EDL muscles throughout the early postnatal development stages. In myoblast cells, shRNA knockdown of *PPARGC1A* significantly inhibited some muscle development and transition-related genes, including *PPP3CA*, *MEF2C*, and *SM* (*P* < 0.01 or *P* < 0.05), and significantly upregulated the expression of *FWM* (*P* < 0.05). Our study demonstrates strong transcriptome differences between oxidative and glycolytic myofibers, and the results suggest that *PPARGC1A* is a key gene involved in chicken myofiber composition and transition.

## Introduction

The Qingyuan partridge chicken is an important indigenous breed in China, and is popular for its superior meat quality [1]. It is a light-body type breed, which is famous for its three “yellow”, two “thin” and one “partridge” morphology features, i.e. yellow beak, shanks and skin; thin head and bone; partridge feather. This breed has high oxidative metabolism leading to desirable traits in muscles, such as higher red muscle ratio, favourable meat colour, marbling, and flavour [2].

Research in recent years has led to remarkable progress in various aspects of poultry breeding, including growth rate, meat yield, and body composition traits, but some meat quality traits such as intramuscular fat (IMF) content, tenderness, water-holding capacity, and colour have been negatively affected [3]. Meat quality is a complex trait and is influenced by many factors, making it difficult to predict and improve [4]. Skeletal muscle, the main tissue determining meat quality and production in chickens, is a heterogeneous tissue composed of different fiber types, varying in their morphology, metabolism, and physiology [5, 6]. Previous studies have found that different myofiber types can influence meat quality [7, 8]. In chickens, myofibers can be divided into oxidative (type I and IIa, red) and glycolytic (type IIb, white). In the chicken, the *soleus* is an oxidative muscle with numerous capillaries and high lipid, myoglobin, and mitochondria content, while the *extensor digitorum longus* and lateral *gastrocnemius* are glycolytic muscles exhibiting fewer capillaries and lower lipids myoglobin, and mitochondria content [9]. A higher content of oxidative (red) fibers in muscles can result in higher meat quality, and there may be a large number of genes taking part in the determination of meat quality [10, 11]. Therefore, it is important to understand the molecular processes that govern the specific myofiber type expression and the phenotypic characteristics of skeletal muscle in the genetic improvement of chicken meat quality.

Microarray technology can identify a large number of differentially expressed genes in a given tissue simultaneously and has been conducted to compare gene expression profiles responsible for relevant phenotypes in animals [12–16]. Previous studies have been compared differences in gene expression between oxidative and glycolytic muscles in various species. Campbell et al. (2001) used Affymetrix Mu11K SubB to discover differentially expressed genes between the white *quad* and the red *soleus* muscle of female mice, and identified 49 differentially expressed genes [17]. Bai et al. (2003) found many candidate genes, which determine muscle red/white phenotype in Berkshire pigs [18]. Li et al. (2010) found 28 signalling pathways, including MAPK and Wnt pathways, which responded to metabolic differences between muscle types in Meishan pigs using Affymetrix Porcine Genechip [19].Zhu et al. (2015) revealed 561 differentially expressed genes between extensor digitorum longus and soleus muscles of large white pigs by RNA-seq technology, and identified many muscle-related genes and pathways related to myofiber formation [20]. Zhang et al. (2015) revealed 168 DEGs between leg muscle (LM) and pectoral muscle (PM) in ducks [21]. However, in chickens, the comparison of global gene expression patterns between muscle fiber types are lacking. Only one report has compared the differences between broiler and layer skeletal muscle cells [22]. In the present study, global differentially expressed genes between oxidative (*soleus*) and glycolytic (*extensor digitorum longus*) muscle of Qingyuan partridge chickens were investigated using the Agilent Chicken Gene Expression Chip (4×44K, Design ID: 026441). The candidate gene *PPARGC1A* was selected for further investigation of its potential involvement in chicken myofiber composition. We aimed to investigate the distinct properties between oxidative and glycolytic myofibers, and we expect that it will provide useful information in improving and controlling chicken meat quality.

## Materials and methods

### Ethics statement

All animal experiments described in the present study were performed in accordance with the guidelines for Experimental Animals established by the Ministry of Science and Technology (2006, Beijing, China). All protocols and procedures were approved by Institution Animal Care and Use Committee in Poultry Institute, Chinese Academy of Agricultural Science, Yangzhou, China. All efforts were made to minimize animal suffering.

### Animals and muscle sampling

Qingyuan partridge chickens (QY, Guangdong Tiannong Food Ltd, Guangdong, China) were used for microarray analysis in this study. Birds were reared in stairstep caging under continuous lighting using standard conditions of temperature, humidity and ventilation. The starter ration (d 1 to d 21) with 20% crude protein and 2.87 MC/kg differed only slightly from that used in the grower (after d 22) phase; 19% crude protein and 3.0 MC/Kg. Feed and water were provided *ad libitum* during the experiment. Three female chickens from a full-sib family of a preserved population of with average body weight 1330 g were slaughtered at 112 days of age (at sexual maturity). Chickens were killed by stunning followed by exsanguination. Two types of muscle from different locations were sampled immediately after slaughter: the *soleus* muscles (SOL) and the *extensor digitorum longus* muscles (EDL). A 1 × 1 cm^2^ section in the middle of the right SOL and EDL was selected, immediately frozen in liquid nitrogen (−160°C), and stored at –80°C. Measurement of the myofiber characteristics including cross-sectional area (CSA), density, size, and myofiber ratios was carried out using mATPase staining. The same portion of the left SOL and EDL muscles were also sampled and immediately frozen in liquid nitrogen, then stored at –80°C prior to RNA isolation.

Ten female individuals were sampled at different developmental stages after hatching (0, 1, 3, 5, 7, and 9 weeks), and SOL and EDL muscles were collected for the gene expression analysis of *PPARGC1A*.

### Measurements of myofiber characteristics

Serial tissue sections of 12 mm thickness were prepared and myosin ATPase staining was used to identify the myofiber characteristics. These were carried out according to our previous report [23].

### Extraction of RNA

Total RNA from SOL and EDL muscles and primary embryonic myoblasts was extracted using Trizol reagent (Tiangen, China). RNA was purified and DNase-treated using an RNeasy^®^ Mini Kit (QIAGEN) according to the manufacturer’s instructions. The RNA quantity of each sample was examined using a NanoDrop ND-2000 spectrophotometer (Thermo Scientific) at 260/280 nm (ratio > 2.0). The integrity of total RNA was analysed with the Agilent Bioanalyzer 2100 and RNA 6000 Nano LabChip Kit (Agilent Technologies) with RIN number > 9.

### Microarray analyses

Microarray hybridization was carried out by Shanghai OE Biotech Limited Company (China) using Agilent Chicken Gene Chips (4×44K, ID: 026441) with 43803 probes. The DEGs were selected out by using Significance Analysis of Microarrays (SAM) software with the following screening criteria: *P* ≤ 0.05; with a fold change ≥ 2; or a fold change ≤ 0.5. The gene ontology enrichment analysis was performed for function corresponding to DEGs in chicken using the GOEAST software toolkit (*P* ≤ 0.05), and signalling pathway analysis was carried out using KEGG data software. Finally, enrichment analyses of DEGs were performed by using the DAVID 6.7 software.

### qRT-PCR analysis

To validate the microarray hybridization results, seven genes were selected from the DEG list for qRT-PCR assays, the primer sequences were listed in S1 Table. All RNA samples used in gene-chip hybridization were also detected in qRT-PCR. For the *PPARGC1A* expression in different muscles and developmental stages in Qingyuan partridge chickens, total RNA was reverse-transcribed using a PrimeScript RT Reagent Kit (Takara Co., Japan). qRT-PCR reactions were carried out using the following thermal profile: after an initial denaturation at 94°C for 2 min, amplification was performed with 40 cycles of 94°C for 30 s, followed by annealing for 40 s at temperatures specific for each target gene. For each sample, reactions were set up in triplicate to ensure the reproducibility of the results. At the end of the PCR run, melting curves were generated and analysed to confirm non-specific amplification, and the mean value of each triplicate was used for further calculations. To calculate the mRNA expression of selected genes, ΔCt values were used for detection of mRNA related to internal control GAPDH expression using the 2^-ΔΔCt^ method. Pearson’s correlation coefficient was further calculated for each gene on the normalized data to quantify the consistency between microarray experiments and qRT-PCR.

### Statistical analysis

The comparison data between the two myofiber types were analysed using one-way analysis of variance (ANOVA, SPSS 17.0). Data are represented as means ± standard error; differences between groups were considered statistically significant at *P* < 0.05.

### shRNA design and transfection

shRNA molecules were synthesized by Shanghai GenePharma Co. Ltd (Shanghai, China) with commercial service. Three shRNAs, each targeting different regions of chicken *PPARGC1A* gene were synthesized. The target sequences in chicken *PPARGC1A* gene were as follows: shRNA-1 (5′-GGACTTCACCTAAGCGAAGT-3′), shRNA-2 (5′-GCAGGGATCCCAAGGTAATAA-3′), and shRNA-3 (5′-GCTCTAGAT CAAGGTCCTTTC-3′). A NC shRNA fragment was produced as the control: 5′- TTCTCCGAACGTGTCACGTTTC-3′.

Primary embryonic myoblasts were prepared from 10-day-old Qingyuan partridge chicken embryos as described by Li et al. (2010)[24]. The concentration of the cells was adjusted to 5×10^6^ cells/ml with DMEM containing 20% FBS. The cells were then seeded in 6-well plates and cultured in a 5% CO_2_ incubator at 37°C. When the cells had grown to approximately 70–80% confluence, myoblasts were transfected with the three shRNAs (*PPARGC1A*-shRNA-1, *PPARGC1A*-shRNA-2, *PPARGC1A*-shRNA-3) and the NC control (*PPARGC1A*-shControl), respectively, at a multiplicity of infection (MOI) of 10, followed by the addition of 2.5 μg/ml polybrene to the medium to improve the efficiency of transfection. Efficiency of transfection was observed at 48 h by counting the ratio of fluorescence cells, and cells were harvested at 72 h, and RNA was extracted from cells to perform qPCR. The expression of *PPARGC1A* gene in cells transfected with different shRNA was compared to that transfected with NC shRNA. Subsequently, we found that *PPARGC1A*-shRNA-2 had the highest transfection efficiency, and we used this for the further transfecting procedures. RNA was collected at 96 h after transfection, and then change in mRNA expression level was analysed for skeletal muscle development and transition-related genes, including *SM*, *FRM*, *MEF2C*, *NFATC3*, and *PPP3CA* (S1 Table).

## Results

### Comparison of myofiber characteristics between SOL and EDL muscles

**Fig 1:**
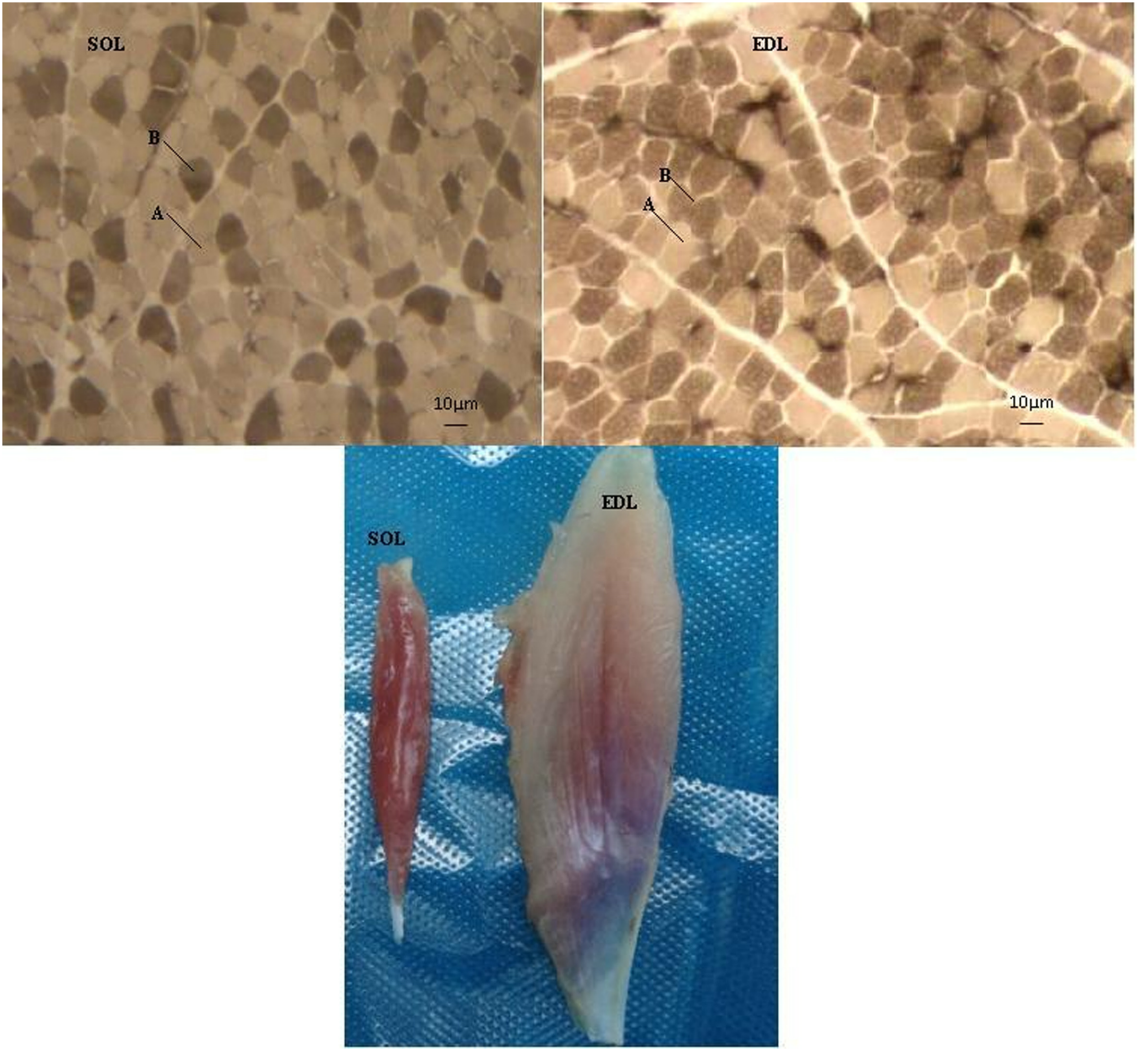
Morphological and fiber-type variations in SOL and EDL muscle samples of Qingyuan partridge chickens. A: white fiber, B: red fiber; Bar: 10 μm.

Compared with *soleus* muscle (SOL), *extensor digitorum longus* (EDL) exhibited a significantly higher cross-sectional area (CSA), diameter, and white myofiber ratio (*P* < 0.05) (Table 1, Fig 1), suggesting that there may be a disparity in the molecular mechanisms behind these differences.

**Table 1:** Myofiber characteristics of the SOL and EDL muscles in Qingyuan partridge chickens.

Myofiber characteristics	SOL^1^	EDL^1^	Sig.^2^
CSA (μm^2^)	1991±112	2863±331	*
Diameter (μm)	47.8±1.3	57.9±4.2	*
Density (number of fibers per μm^2^)	428±23	309±43	*
Red myofiber ratio	0.79±0.02	0.15±0.04	*
White myofiber ratio	0.21±0.02	0.85±0.04	*

^1^ SOL, EDL represent *soleus* muscles and *extensor digitorum longus* muscles, respectively

^2^ Significance: * (*P* < 0.05)

### Identification of differentially expressed genes between SOL and EDL muscles

In the present study, 43,803 probes were used to detect mRNA expression profiles in chicken SOL and EDL muscles. Of these, probes displaying hybridization signals represented 80.7–93.7% of the total (detected value: *P* < 0.05), and the remaining genes lacked hybridization signals (0.05 < *P* < 0.065) or showed ambiguous hybridization signals (*P* > 0.065) (Table 2). The microarray assay data discussed in this publication have been submitted to NCBI’s Gene Expression Omnibus under series accession number GSE69918.

**Table 2:** Summary of gene expression in SOL and EDL muscles in Qingyuan partridge chickens as determined by microarray analysis.

Hybridization signals	*SOL*1	*SOL*2	*SOL*3	*EDL*1	*EDL*2	*EDL*3
Present Probes	38093	39256	38233	41043	37239	35344
Absent Probes	5709	4544	5567	2760	6564	8454
Marginal Probes	1	3	3	0	0	5
Total probes	43803	43803	43803	43803	43803	43803

In total, 1224 genes with at least 2-fold differences were identified at the *P* < 0.05 significance level (*P* < 0.05, FC ≥ 2), out of which 654 genes were upregulated and 570 genes were downregulated when comparing SOL to EDL (S2 Table). The variably expressed genes were involved in many functions related to contractile structure and cytoskeleton, cell signalling, energy metabolism, stress, transcription regulation, fatty acid synthesis and metabolism, and others (Table 3).

**Table 3:** List of some differentially expressed genes between red and white muscle of Qingyuan partridge chickens.

Gene title	Fold change	P value	Structure and function	Unigene
**Muscle contraction and cytoskeleton**				
myosin heavy chain 7B (*MYH7B*)	1204.8	0.0011	striated muscle contraction, actin binding	Gga.103
myosin light chain 2 (*MYL2*)	141.5	0.0055	abnormal cardiac muscle contractility	Gga.841
fast myosin heavy chain (*MYH1E*)	−57.5	0.0006	muscle contraction	Gga.51379
myosin binding protein H (*MYBPH*)	−5.0	0.0322	bind to myosin	Gga.882
myosin binding protein C, fast type isoform 2 (*MYBPC2*)	−3.8	0.0085	bind to myosin	Gga.4986
myosin binding protein C, slow type isoform 1 (*MYBPC1*)	3.5	0.0060	bind to myosin	Gga.10173
slow myosin heavy chain 1 (*SM1*)	4.5	0.0054	muscle contraction	Gga.16803
cysteine and glycine-rich protein 3 (*CSRP3*)	56.5	0.0275	Enlarged myocardial fiber	Gga.5554
**Transcription factor**				
peroxisome proliferator-activated receptor alpha (*PPARA*)	4.1	0.0019	positive regulation of transcription	Gga.4006
peroxisome proliferator-activated receptor gamma coactivator 1-alpha (*PPARGC1A*)	2.2	0.0001	positive regulation of transcription	Gga.17979
peroxisome proliferator-activated receptor gamma, coactivator 1 beta (*PPARGC1B*)	2.2	0.0014	positive regulation of transcription	
general transcription factor IIIC, polypeptide 6, alpha 35 kDa (*GTF3C6*)	−2.2	0.0193	regulation of transcription initiation	Gga.22474
Homeobox protein Hox-A2 (*HOXA1*)	−3.6	0.0006	regulation of transcription	Gga.55802
Epidermal growth factor receptor kinase substrate 8-like protein 2 (*EPS8L2*)	2.1	0.006	calcium ion binding, integral to membrane	
**Cell signalling**				
protein phosphatase 2, catalytic subunit, alpha isozyme (*PPP2CA*)	3.6	0.0009	Regulation of cell signalling	Gga.8891
protein phosphatase 3, catalytic subunit, alpha isozyme (*PPP3CA*)	−3.6	0.0234	Regulation of cell signalling	Gga.48561
protein phosphatase 3, regulatory subunit B, alpha (*PPP3R1*)	-2.0	0.0200	Regulation of cell signalling	Gga.3833
vascular endothelial growth factor A (*VEGFA*)	2.9	0.0143	Regulation of growth retardation	Gga.537
fibronectin type III domain containing 5 (*FNDC5*)	4.5	0.0091	extracellular region	Gga.6248
Collagen, type I, alpha 1	−4.2	0.0439	phosphate transport, cell adhesion	Gga.2073
collagen, type IX, alpha 3 (*COL9A3*)	3.8	0.02	phosphate transport, cell adhesion	Gga.3459
collagen, type III, alpha 1 (*COL3A1*)	−3.1	0.0228	phosphate transport, cell adhesion	Gga.42140
**Metabolic enzyme**				
protein kinase, AMP-activated, gamma 3 non-catalytic subunit (*PRKAG3*)	−10.8	0.0017	AMP-activated protein kinase activity	Gga.22949
calpain 3, (p94) (*CAPN3*)	−2.1	0.0362	calcium-dependent cysteine-type endopeptidase activity	Gga.72
pyruvate dehydrogenase kinase, isozyme 1 (*PDK1*)	4.7	0.0003	phosphorylate pyruvate dehydrogenase	Gga.21396
heme oxygenase 1 (*HMOX1*)	2.2	0.0378	heme oxidation	Gga.2039
phosphoglucomutase 1 (*PGM1*)	−3.7	0.024	phosphotransferases, carbohydrate metabolic process	Gga.33728
creatine kinase, mitochondrial 1A (*CKMT1A*)	−2.7	0.0011	transferring phosphorus-containing groups	Gga.13490
**Fatty acid synthesis and metabolism**				
stearoyl-CoA desaturase 5 (*SCD5*)	2.2	0.0069	Fatty acid synthesis	Gga.41967
fatty acid synthase (*FASN*)	−2.3	0.0000	fatty acid synthesis	Gga.5501
lipoprotein lipase (*LPL*)	3.1	0.0121	fatty acid uptake and transport	Gga.1152
fatty acid binding protein 3, muscle and heart (*FABP3*)	4.1	0.0039	fatty acid uptake and transport	Gga.12266

“+” and “−” indicates up or downregulated expression in the *soleus* group.

### Validation of gene expression changes by qRT-PCR

**Fig 2:**
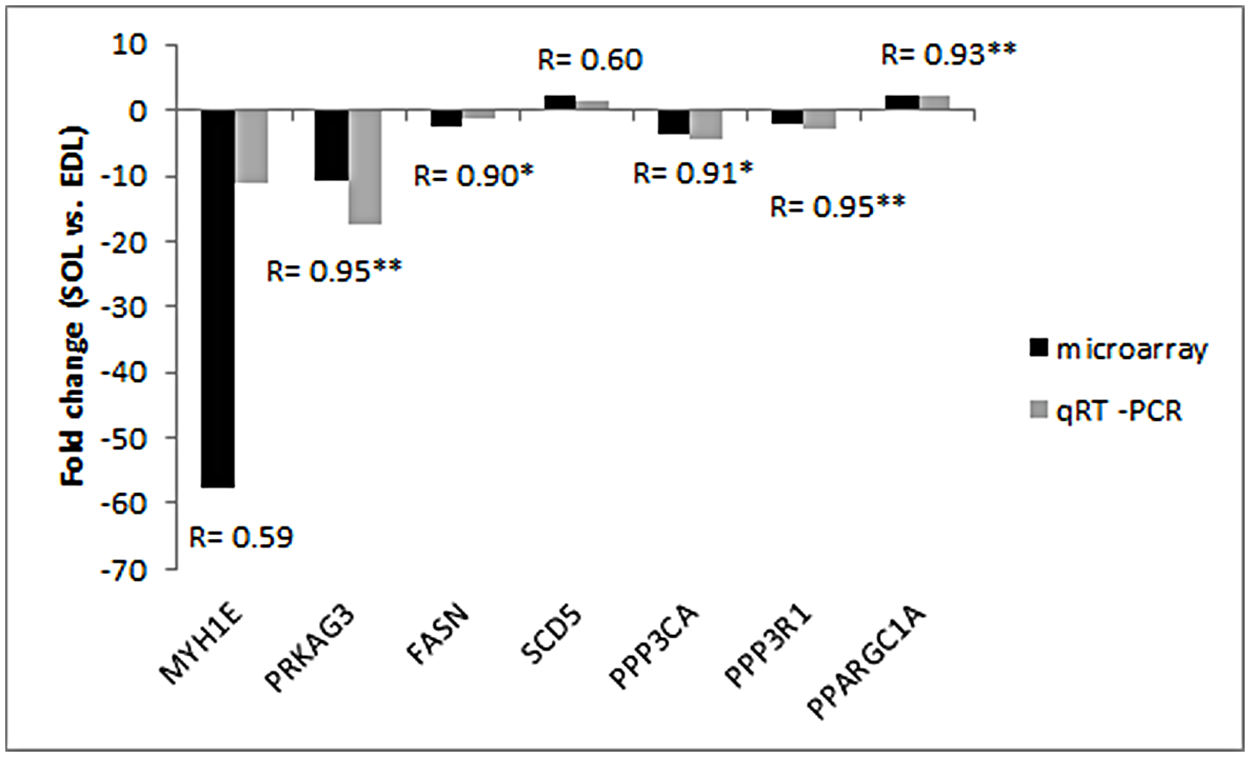
Difference of fold change in mRNA level of each gene between microarray and qRT-PCR analysis. Fold changes were calculated as mRNA levels in SOL compared with EDL. Bars above X-axis indicate that genes were highly expressed in SOL, whereas those under X-axis indicate that genes were highly expressed in EDL. R indicates the Pearson correlation coefficient. * indicates significance level at *P* < 0.05; ** indicates significance level at *P* < 0.01.

Seven known genes were randomly selected from the variably expressed genes for validation by qRT-PCR. These included five downregulated genes (*MYH1E*, *PPP3CA*, *PPP3R1*, *PRKAG3*, and *FASN*) and two upregulated genes (*PPARGC1A* and *SCD5*) in SOL. With the exception of *FASN*, all selected genes showed significant differences in expression between the two muscles (*P* < 0.05 or *P* < 0.01) (S1 Fig). Remarkably, all the genes showed similar expression patterns using both methods, and positive correlation between the two methods was found using a Pearson correlation coefficient ranging from 0.59 to 0.95. These results confirm the reliability of the microarray analysis (Fig 2).

### GO and KEGG pathway analysis for DEGs

A total of 1224 significantly different genes were mapped to the Gene Ontology database (geneontology.org), and 74 significantly different GO terms were obtained (*P* < 0.05). These terms were categorized into three groups: biological processes (34 GO terms), molecular functions (20 GO terms), and cellular components (20 GO terms) (S3 Table). According to Gene Ontology, genes that were variably expressed between SOL and EDL and participated in biological process terms were mainly focused on energy metabolism (i.e. small molecule metabolism, carbohydrate metabolism, glycolysis, redox reactions, steroid metabolism, and pyruvate metabolism). Three processes related to muscle development were observed, including transition between fast and slow fiber, embryonic skeletal joint morphogenesis, and skeletal muscle contraction. Six DEGs were found to be involved in these processes: *TNNC1*, *ATP2A2*, *HOXD11*, *COL2A1*, *CHRNA1*, and *TNNI2*. These genes may be crucial to muscle composition and development.

**Fig 3:**
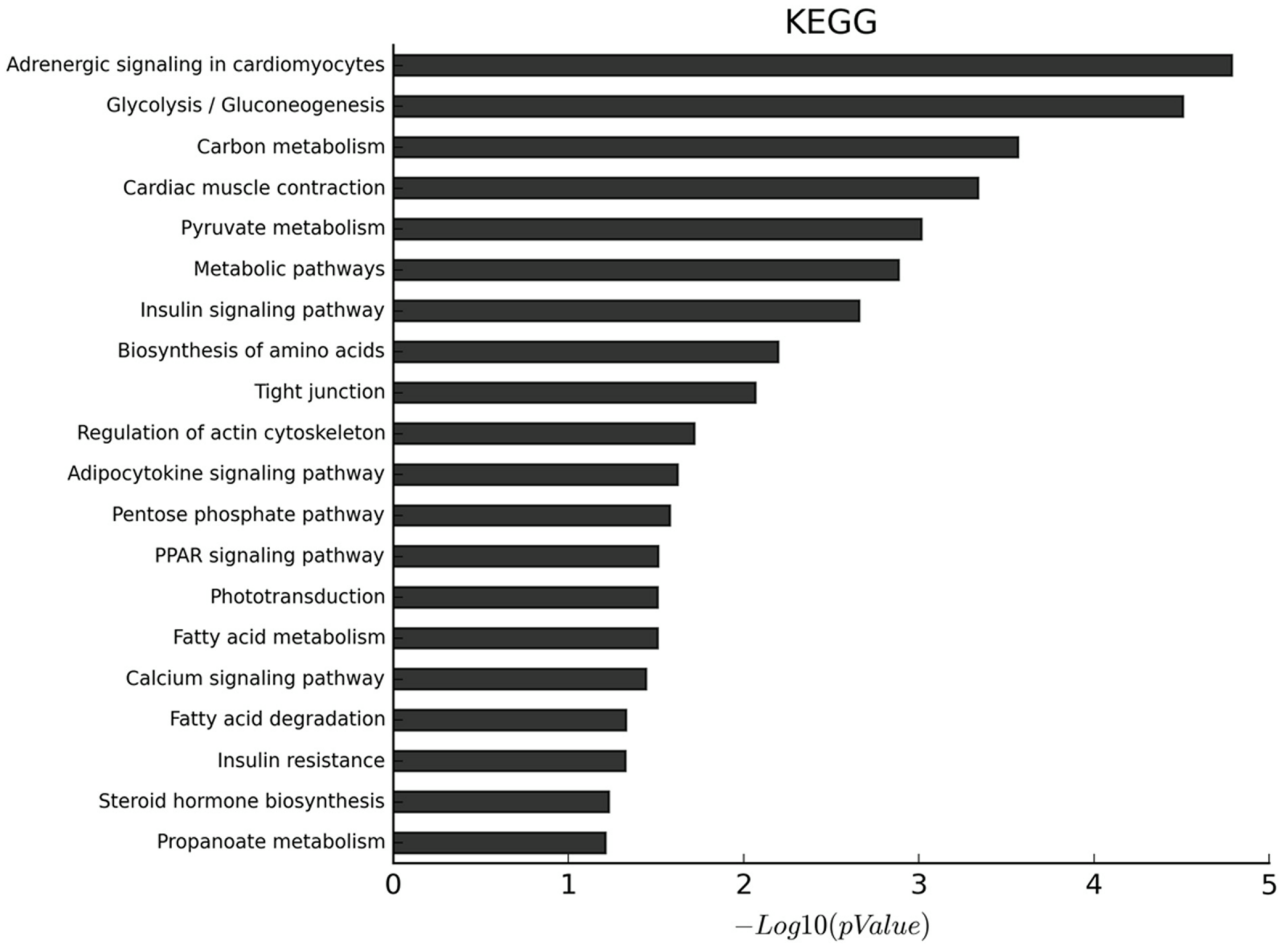
Enriched KEGG pathway for all DEGs identified between SOL and EDL muscles.

The identified DEGs were significantly enriched in twenty KEGG pathways (*P* < 0.05), with the most influenced pathway being adrenergic signalling in cardiomyocytes (Fig 3). Well-known pathways affecting muscle fiber transition (calcium and PPAR signalling), muscle development (insulin signalling) and lipid metabolism (adipocytokine- signalling) were enriched in both types of muscles (S4 Table). There was 62 DEGs in these four pathways, including ATP2A2, PRKAG3, PPARGC1A, and TNNC1.

### Coexpressed gene network for DEGs

**Fig 4:**
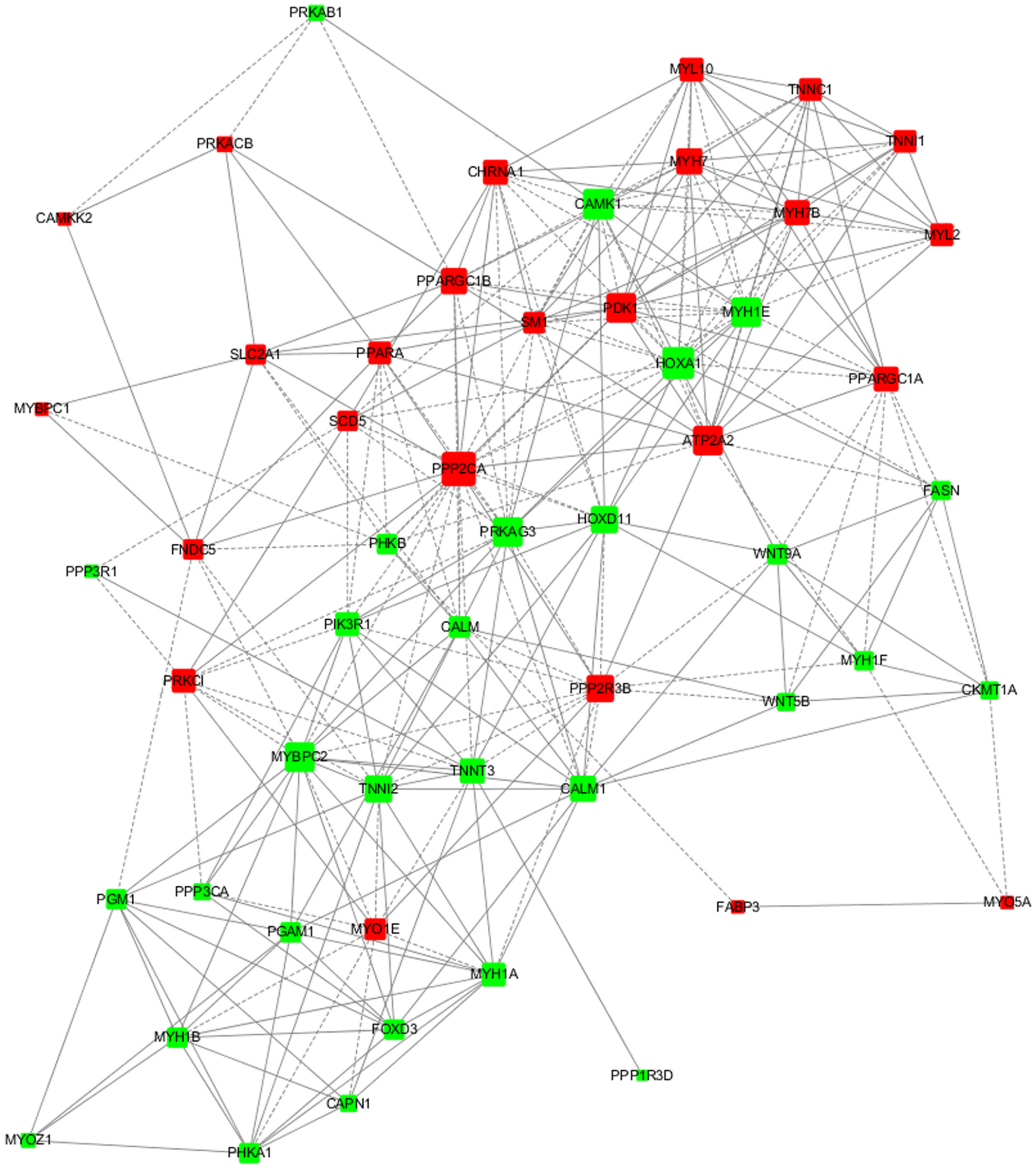
Coexpressed gene network for the selected DEGs. Genes exhibiting upregulation are shown in red, whereas genes exhibiting downregulation are shown in green. The colour intensity indicates the degree of up/downregulation. Solid lines and dashed lines indicate positive correlation and negative correlation, respectively.

Subsequently, Pearson’s correlation analysis was used to investigate the gene correlations for some known DEGs related to muscle development and composition according to the previous reports [17–22]. Three hundred mRNA-mRNA couples with r^2^ > 0.8 and *P* < 0.05 were selected, and Cytoscape 3.3 software was used to construct gene networks. The results showed that *PPP2CA*, *PRKAG3*, *ATP2A2*, *PPARGC1A*, and *PPARGC1B* were node genes (Fig 4).

The results of the GO, KEGG pathway, and gene coexpression network analyses indicated that *PRKAG3*, *ATP2A2*, and *PPARGC1A* might be key genes that determine chicken muscle fiber characteristics. *ATP2A2* and *PPARGC1A* mRNA levels were higher in oxidative muscle than in glycolytic muscle. In the present study, we chose *PPARGC1A* for further functional analysis.

### *PPARGC1A* mRNA expression in SOL and EDL tissues in postnatal Qingyuan partridge chickens

**Fig 5:**
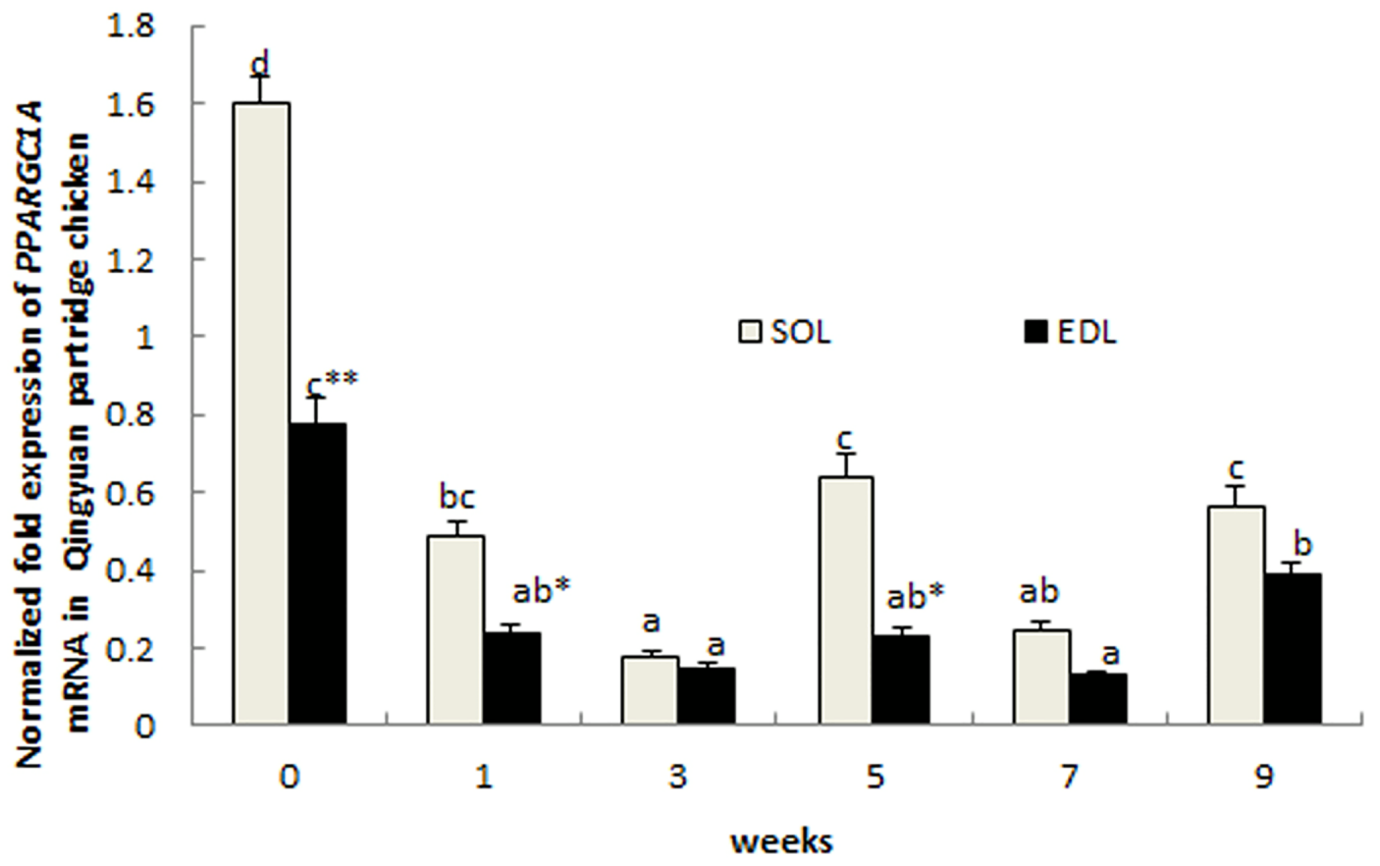
*PPARGC1A* gene expression levels in SOL and EDL muscles in Qingyuan partridge chickens during early post-hatching development. ** and * indicated *P* < 0.01 and *P* < 0.05, respectively. Bar diagram values with the same letter are not significantly different among different ages in the same tissue (*P* > 0.05), and values with different letters are significantly different between different ages in the same tissue (*P* < 0.05).

Throughout early development of Qingyuan partridge chicks, *PPARGC1A* mRNA expression exhibited a similar pattern in the SOL and EDL tissues. Expression level was the highest at birth (week 0), then decreased significantly from week 1 to week 3 (*P* < 0.05), followed by an increase from week 3 to week 5, then a decrease from week 5 to week 7, and finally a significant elevation at week 9 (*P* < 0.05) (Fig 5). The expression of *PPARGC1A* was consistently higher in SOL muscle than in EDL muscle throughout development, with significant differences observed at 0, 1, and 5 weeks (P >0.05).

### Effect of *PPARGC1A*-shRNA-2 recombinant virus on expression of myofiber-related genes

**Fig 6:**
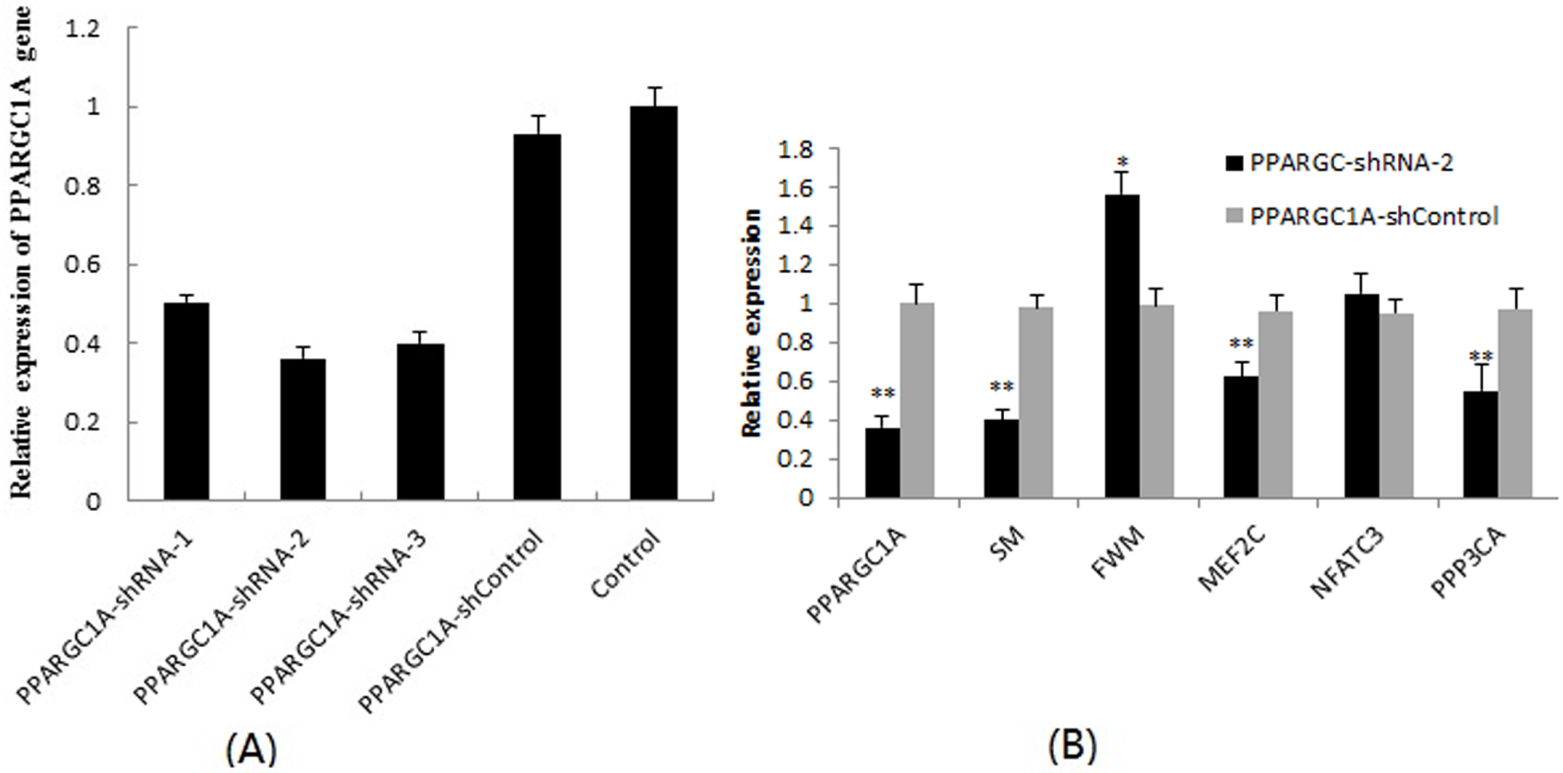
*PPARGC1A* expression in myoblasts and the up/downregulation of myofiber-related genes. (A) Relative expression of *PPARGC1A* in myoblasts transfected with different shRNAs; (B) Relative expression changes of myofiber-related genes in myoblasts transfected with shRNA-1317. **and*indicated *P* < 0.01 and *P* < 0.05, respectively.

The three shRNAs (*PPARGC1A*-shRNA-1, *PPARGC1A*-shRNA-2, and *PPARGC1A*-shRNA-3) decreased PPARGC1A gene expression by 49.7%, 63.7%, and 59.9%, respectively (Fig 6A). Therefore, *PPARGC1A*-shRNA-2 was used for transfecting myoblast cells. At 96 h after transfection, qRT-PCR results showed an effective inhibition of *PPARGC1A* expression (*P* < 0.01) (Fig 6B). Moreover, knockdown of *PPARGC1A* expression resulted in significant downregulation of some muscle development and transition related genes, including *PPP3CA* (*P* < 0.01), *MEF2C* (*P* < 0.05), and *SM* (*P* < 0.01), whereas *FWM* expression was significantly upregulated (*P* < 0.05), and the expression change of *NFATC3* was not significantly (*P* > 0.05) (Fig 6B).

## Discussion

The Qingyuan partridge chicken is an important indigenous breed in south of China, and consumption habits of the local people like to eat female chickens, so we chose female Qingyuan partridge chickens as research materials in the present study. Muscle fiber composition can influence meat quality traits, including colour, tenderness, water-holding capacity, juiciness, and flavour [7, 8, 25]. A high content of type I (oxidative) fiber contributes more to juiciness and flavour [25]. Our results showed significant phenotypic differences between SOL and EDL muscles in Qingyuan partridge chickens, wherein EDL exhibited a significant higher cross-sectional area (CSA), diameter, and white myofiber ratio than SOL. Although some DEGs between different skeletal muscles have recently been identified in several species, such as mice [17], pigs [18–20], and duck [21], the molecular mechanism underlying muscle fiber characteristics in chicken remains unclear. Therefore, our objective was to clarify the biological events, which could explain phenotypic differences between the SOL and EDL in chicken. In brief, 1224 differentially expressed probes between SOL and EDL were identified, and the fold-change directions of the DEGs observed in microarray analysis have been validated by qRT-PCR analyses. The number of genes found differentially expressed between oxidative and glycolytic muscles is relatively high, when compared with those DEGs found in pigs and ducks. There were 159 DEGs between red (soleus) and white muscles (extensor digitorum longus) in Chinese Meishan pigs [19], 561 DEGs between red (soleus) and white muscles (extensor digitorum longus) in large white pigs [20], and 168 DEGs between leg muscle and pectoralmuscle in ducks [21]. However, different experimental conditions such as species, sample size, technology platform, FC threshold might account for this discrepancy.

### Function of DEGs implicated in muscle development and composition

The diversity in muscle fiber expression and accumulation of contractile proteins in adult animals appears to be directed by neural activity [26], so genes that are related to muscle development, energy metabolism, lipid metabolism or nerve activity would contribute to the differentiation and maturation of skeletal muscle fibers. In the present study, There are 14 DEGs (*MYH7B*, *CSRP3*, *TNNT2*, *RSPO3*, *MYL1*, *MYBPC1*, *PRSS35*, *P4HA3*, *XPO4*, *FREM2*, *FBP1*, *SMTNL2*, *SMYD1*, *FAT3*) were also found between oxidative and glycolytic muscles in both pigs [19, 20] and ducks [21], these genes might be related to skeletal myofiber composition. Moreover, several DEGs were also been previously studied in the other species, such as *PGM1*, *fibronectin*, *HMOX1*, *SM1*, *MYH1E*, *PPARGC1A*, *PPARGC1B*, *PPP3CA*, and *PPP3R1* [17, 27–29]. Genes related to slow-type muscle protein encoding (*MYBPC1*, *SM1*), energy metabolism (*PDK1*, *PPARA*) and muscle contraction (*MYH7B*, *MYL2*) which could contribute to better meat quality of red muscle, were more highly expressed in oxidative muscle than in glycolytic muscle. All of these variances in the gene profile can provide explanation for the differences in the fiber types in SOL and EDL muscles.

GO analysis demonstrated that genes involving in energy metabolism and muscle development were over-represented in both type of muscles, consistent with the distinct energy expenditure regulation features among different fiber types [30], and also consistent with the differences found between red and white skeletal muscle of Chinese Meishan pigs [19]. Energy availability plays an important role in the formation, proliferation, and differentiation of mature muscle fibers. Louis et al. (2004) reported that the energy content of cultured satellite cells could affect myofiber hypertrophy in vitro, which indicates that there is a direct relationship between myogenesis and energy metabolism [31]. Cagnazzo et al. (2006) also reported a direct connection between energy metabolism and myogenic differentiation [32].

Based on the functional annotation analysis of DEGs from different muscle tissues, we identified adrenergic signalling in cardiomyocytes was the most significantly enriched pathway. The adrenergic signalling system is an important protein signalling system, which can regulate [Ca^2+^]_i_ transients, cardiac action potential duration, and contraction force [33]. Moreover, other enriched pathways were muscle fiber transition (calcium and PPAR signalling), muscle development (insulin signalling) and lipid metabolism (adipocytokine signalling), suggesting that the regulation of muscle development and composition is possibly affected by the interaction of complex pathway involving muscle, fat and connective tissue. Calcium signalling has been implicated through calcineurin-a calcium-calmodulin (CaM)-dependent protein phosphatase and CaM kinase in the control of type I fiber-specific contractile proteins [34, 35]. PPAR signalling is important in muscle fiber type regulation [36]. Insulin such as IGF-I has long been recognized as having the ability to stimulate the rate of myoblast differentiation [37] and influence myogenin expression [38].

Microarray experiments result in a large number of variably expressed genes, and the further functional data analysis is essential to determine relevant biological interpretations. In this context, some DEGs that were related to muscle development and composition finding in GO and pathway analysis or former literature reports [17–21] were chosen for coexpressed gene network analysis. Importantly, we identified three crucial genes (*PRKAG3*, *ATP2A2*, and *PPARGC1A*) which may be related to chicken myofiber composition. Network analysis indicated that *PRKAG3*, *ATP2A2*, and *PPARGC1A* interacted with each other, consistent with a previous report [39]. PRKAG3 is a –γ3 subunit of AMPK (a heterotrimeric serine/threonine protein kinase), which is exclusively expressed in skeletal muscle. In the present study, *PRKAG3* mRNA levels were significantly higher in EDL than that in SOL muscles (FC = 10.82, *P* < 0.01), which could imply a higher response of the AMPK complex to AMP, which in turn would be consistent with greater amounts of glycogen in muscle. This result was also consistent with the previous reports that γ3 is more highly expressed in fast-twitch glycolytic than oxido-glycolytic fibers, and is even undetectable in slow-twitch oxidative fibers in mammals [40]. *ATP2A2* is a gene encoding a slow skeletal and cardiac muscle-specific Ca2+ ATPase, SERCA2. Wei et al. (2015) reported that *ATP2A2* could regulate slow-twitch muscle gene expression [41]. The highly expressed of *ATP2A2* mRNA level in SOL (FC = 151.9, *P* < 0.001) in the present study also supported this conclusion. The peroxisome proliferator-activated receptor-gamma coactivator-1A (*PPARGC1A*, also known as *PGC-1α*), which was originally identified via its functional interaction with peroxisome proliferator-activated receptor gamma, is an important regulator of many metabolic pathways [27, 42]. Importantly, *PPARGC1A* has been implicated as a principal factor in regulating muscle fiber type determination, and has been shown to drive fast/glycolytic fiber type switching to slow/oxidative fibers [27, 43]. Our previous study investigated the genetic effects of the *PPARGC1A* gene on chicken skeletal muscle fiber characteristics, and found that the polymorphisms of the *PPARGC1A* gene and their haplotypes are associated with chicken skeletal myofiber type traits [2]. Therefore, *PPARGC1A* was chosen for the further functional analysis.

### The function of PPARGC1A gene in muscle fiber composition

*PPARGC1A* mRNA expression patterns in SOL and EDL were further analysed during early postnatal development stages of Qingyuan partridge chickens. Expression levels were higher in SOL than in EDL muscles throughout the studied development. This result was consistent with that of a previous study by Lin et al. (2002) in mice, wherein PGC-1α messenger RNA expression was shown to mimic the expression of troponin I (slow), a classical marker of type I fiber; PGC-1α is highly expressed in *soleus* but has much lower expression in type II-rich muscles such as EDL, *quadriceps*, *tibialis anterior*, and *gastrocnemius* [27]. Moreover, the mRNA expression of *PPARGC1A* exhibited a similar pattern in both SOL and EDL muscles, wherein the highest expression level occurred at hatching, and then decreased significantly to a relatively low level. Muscle fiber formation is completed at hatching in avian, and postnatal muscle growth is determined by myofiber hypertrophy, which is accompanied with myofiber-type transformation following the sequence: SM-FRM-FWM [44]. The highest expression at birth may also indicate that PPARGC1A has some effect on the formation of myofibers.

RNA interference (RNAi) mediated by short hairpin RNAs (shRNAs) has become a powerful tool for gene knockdown studies. To further study the function of *PPARGC1A* gene in myofiber transition, we transfected chicken embryo primary myoblasts with three lentivirus-mediated *PPARGC1A* shRNA constructs and observed 49.7–63.7% reductions in *PPARGC1A* transcript levels. The efficiency of transfection and knockdown was relatively high in primary myoblast cells comparing to the similar knockdown studies in myoblast cells [45, 46]. These results demonstrate that lentivirus-mediated *PPARGC1A* shRNA can successfully silence *PPARGC1A* gene expression in chicken embryo myoblasts. Under knockdown, genes involved in calcium signalling such as *PPP3CA* and *MEF2C* and the MyHC SM isoform were significantly downregulated, whereas the MyHC FRM isoform was significantly upregulated, suggesting that *PPARGC1A* may play an important role in chicken myofiber composition and can co-activate the transcriptional activity of calcium signalling genes. Calcium signalling is a chief regulatory pathway of type I fiber-selective gene expression [47]. Our results also supported the previous report that cooperation between *PPARGC1A* and the calcineurin pathway is probably critically important in myofiber type switching, and a significant portion of this interaction may occur as a consequence of the direct co-activation of Mef2 proteins by *PPARGC1A* [27].

## Conclusions

The present study provided a global transcriptome analysis between oxidative (SOL) and glycolytic (EDL) muscles in Qingyuan partridge chickens. The results suggest that the identified DEGs are related to the phenotypic differences of these two muscles. *PRKAG3*, *ATP2A2*, and *PPARGC1A* may play key roles in chicken myofiber composition and transition. Further expression analysis and shRNA analysis demonstrated that *PPARGC1A* is a key gene involved in chicken myofiber composition and transition. Our observations provide a basis for further exploration of these molecular processes.

## Supporting information

S1 Fig Relative mRNA expression of the seven selected genes between SOL and EDL by qRT-PCR. * indicates significance level at *P* < 0.05; ** indicates significance level at *P* < 0.01.
(TIF)

S1 Table Descriptions of all primers used in this study. (XLS)

S2 Table Detailed information on DEGs between SOL and EDL in Qingyuang partridge chickens. (XLS)

S3 Table The GO category for DEGs between SOL and EDL in Qingyuang partridge chickens. (XLS)

S4 Table Identification of enriched KEGG pathways based on the DEGs between SOL and EDL in Qingyuang partridge chickens. (XLS)

## Abbreviations

ATP2A2: ATPase, Ca^2+^ transporting, cardiac muscle, slow twitch 2
CHRNA1: cholinergic receptor, nicotinic, alpha 1 (muscle)
COL2A1: collagen, type II, alpha 1
EDL: *extensor digitorum longus*
FASN: fatty acid synthase
FWM: fast myosin heavy chain
HOXD11: homeobox D11
IMF: intramuscular fat
MEF2C: myocyte enhancer factor 2C
MYH1E: fast myosin heavy chain
NFATC3: nuclear factor of activated T-cells, cytoplasmic calcineurin-dependent 3
PPARGC1A: peroxisome proliferator-activated receptor gamma coactivator 1-alpha
PPARGC1B: peroxisome proliferator-activated receptor gamma, coactivator 1 beta
PPP2CA: protein phosphatase 2, catalytic subunit, alpha isozyme
PPP3CA: protein phosphatase 3, catalytic subunit
PPP3R1: protein phosphatase 3, regulatory subunit B
PRKAG3: protein kinase, AMP-activated, gamma 3 non-catalytic subunit
SCD5: stearoyl-CoA desaturase 5
SM: slow myosin heavy chain
SOL: *soleus*
TNNC1: troponin C type 1 (slow)
TNNI2: troponin I type 2 (skeletal, fast)
