# Supplementary figures and images for: Oxidative and glycolytic skeletal muscles show marked differences in gene expression profile in Chinese Qingyuan partridge chickens
Source: PLoS One. 2017 Aug 16;12(8):e0183118. doi: 10.1371/journal.pone.0183118 (PMC5558948; doi:10.1371/journal.pone.0183118)

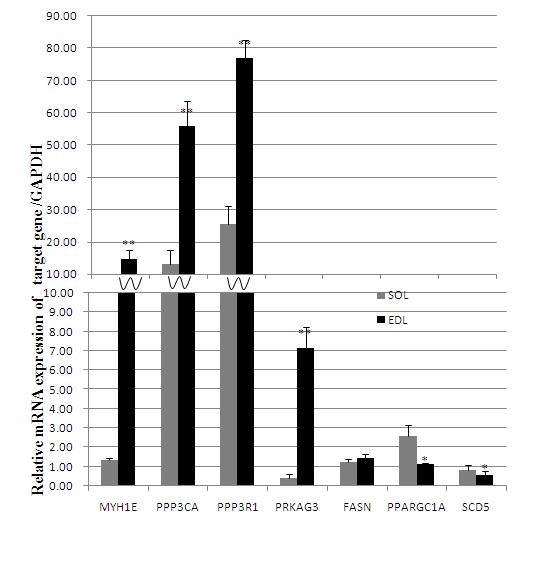

Supplement: S1 Fig — * indicates significance level at P < 0.05; ** indicates significance level at P < 0.01. (TIF) [file pone.0183118.s001.tif]
